# The role of cardiovascular magnetic resonance in takotsubo syndrome

**DOI:** 10.1186/s12968-016-0279-5

**Published:** 2016-10-12

**Authors:** Rui Plácido, Bernardo Cunha Lopes, Ana G. Almeida, Carlos E. Rochitte

**Affiliations:** 1 Cardiovascular Magnetic Resonance and Computed Tomography Sector, Heart Institute, InCor, University of São Paulo Medical School, São Paulo, Brazil; 2 Cardiology Department, Lisbon Academic Medical Centre, CCUL, Santa Maria University Hospital, Lisbon, Portugal

**Keywords:** Cardiovascular magnetic resonance, Takotsubo syndrome

## Abstract

Takotsubo syndrome (TS) is a transient form of left ventricular dysfunction associated with a distinctive contraction pattern in the absence of significant coronary artery disease triggered by stressful events. Several aspects of its clinical profile have been described but it still remains difficult to quickly establish the diagnosis at admission.

Cardiovascular magnetic resonance (CMR) has achieved great improvements in the last years, which in turn has made this imaging technology more attractive in the diagnosis and evaluation of TS. With its superior tissue resolution and dynamic imaging capabilities, CMR is currently the most useful imaging technique in this setting.

In this review, we propose to comprehensively define the role of CMR in the evaluation of patients with TS and to summarize a set of criteria suitable for diagnostic decision making in this clinical setting.

**Electronic supplementary material:**

The online version of this article (doi:10.1186/s12968-016-0279-5) contains supplementary material, which is available to authorized users.

## Background

Takotsubo syndrome (TS) is a clinical condition that was firstly described in 1990 by Sato et al*. *[1], featuring a reversible left ventricular (LV) dysfunction with symptoms similar to those of acute coronary syndromes typically without significant epicardial coronary lesions [2]. The defining hallmark of this entity is the recovery of function occurring within days to weeks of the index clinical presentation.

The precise incidence of TS is unknown, but studies revealed a prevalence of 1–2 % of patients presenting with suspected acute coronary syndromes [3].

The pathophysiological origin for this syndrome remains elusive and different etiologies are currently considered, with an excess of catecholamines precipitated by a situation of stress being of note. Activation of the sympathetic nervous system precipitates cardiac adrenergic stimulation and a subsequent change in contractility and electrophysiological status of the myocardium [4]. Specifically, cardiac adrenergic dysfunction characterizes the acute phase of the syndrome with gradual normalization during the following weeks or months.

TS has generally been regarded as a relatively benign disease [5]. However, it is characterized by substantial morbidity and mortality [6], and complications are more frequent than previously thought, ocurring in up to 50 % of patients [7–9].

Advanced imaging modalities are becoming increasingly important in cardiovascular disease management, providing essential tools towards the diagnostic and prognostic work-up of patients with TS. Excluding significant coronary stenosis is part of its diagnostic algorithm. This evaluation is most often done invasively, as the patient usually presents with a clinical picture closely resembling that of acute myocardial infarction, but could be performed by coronary computed tomography angiography in selected cases.

**Fig. 1:**
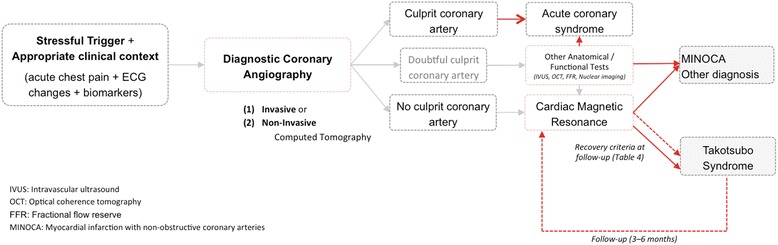
Recommended diagnostic algorithm for takotsubo syndrome

Cardiovascular magnetic resonance (CMR) has become a primary tool for non-invasive assessment of patients with TS (Fig. 1), offering a unique combination of safety, detailed anatomical visualization and tissue characterization data, with inter-observer consistency and quantitative accuracy.

In this review we propose to explore the utility of CMR in the evaluation of patients with TS.

## Cardiovascular magnetic resonance

During the recent decade, CMR has become the diagnostic tool of choice in tertiary care centers for patients with evidence of acute nonischemic myocardial injury. CMR has the unique ability to noninvasively demonstrate myocardial tissue injury through the presence of edema and/or delayed gadolinium contrast washout in myocardial extracellular matrix, providing distinctive insights into the pathogenesis and tissue pathology of several cardiac conditions.

As experience and knowledge of TS has increased, the proposed Mayo diagnostic criteria for the syndrome have evolved [10]. These criteria highlight the essential role of CMR, not only for morphological characterization but also for the exclusion of other entities that otherwise would not be ruled out, particularly myocarditis and myocardial infarction with non-obstructive coronary arteries (MINOCA), a heterogeneous entity with many potential etiologies.

Table 1 summarizes the main applications of CMR in patients with suspected TS. So far, various CMR criteria have been used in several small groups of TS as well as in relatively large population [11].

**Table 1:** Applications of CMR in patients with suspected TS

Qualitative and quantitative assessment of regional wall motion abnormalities.	
Precise quantification of right and left ventricular function.	
Tissue caractherization, providing markers for reversible (inflammation, edema) and irreversible (necrosis, fibrosis) injury.	
Assessment of additional abnormalities – pericardial effusion, pleural effusion and ventricular thrombi.	
Depiction of mechanical complications.	
Differential diagnosis	

### Diagnostic targets and protocol

In a clinical setting for evaluation of TS, CMR appears as the one technique to provide a comprehensive assessment, including accurate and reproducible measurement of biventricular function and volumes, the possibility of serial evaluation, assessment of complications, myocardial perfusion and tissue characterization (Table 1). Cardiac specific sequences are usually implemented in 1.5 T and 3 T scanners. Scanners of 1 T might have available cardiac sequences, and although limited, particularly on the signal-to-noise ratio, could also be used if that is the only available scanner, with potential benefit specially for the patient with claustrophobia or large body size.

Steady state free precession cines, phase contrast sequences, black-blood T2-W triple IR, first-pass perfusion, early gadolinium enhancement (EGE) and late gadolinium enhancement (LGE) sequences are the most widely used sequences in TS. Other CMR techniques such as T1 and T2 mapping techniques and feature/tissue tracking could be useful in the near future though at present limited to research purpose.

A proposed standard CMR protocol in TS is depicted in Table 2.

**Table 2:** Summary of CMR protocol for patients with suspected TS

Protocol	Sequence	Planes	Usefulness and Current Utilization
Scout	Balanced Steady State Free Precession (bSSFP) Non-ECG-gated	Transaxial, coronal and sagittal covering the entire thorax	Standard for all CMR studies Easily identify pleural and pericardial effusions
Edema	Black blood T2-weighted (fast spin echo) triple-inversion recovery (IR)	Short-axis plane covering the LV Slice thickness = 8 mm	Recommended for differential distinction from myocarditis or acute MI. Usual finding is increase SI in mid-apical segments
T1-Mapping	Modified Look-Locker (MOLLI), Shortened Modified Look-Locker Inversion Recovery (ShMOLLI), saturation recovery single shot acquisition (SASHA), others	Short-axis plane covering the LV with specific TI = 100–5000 ms, collected using bSSFP readouts Slice thickness = 8 mm	Research tool that may serve as a complementary technique to T2-weighted imaging. Quantitative means to detect myocardial edema without the need for reference ROIs
T2-Mapping	T2-prepared single-shot SSFP sequence, Multiecho FSE (MEFSE), others	Matching short-axis T1	Application under research evaluation. T2 values more closely correlate with free water tissue content over T1-based techniques in suspected myocardial inflammation. It may offer a more stable and truly quantitative alternative for edema detection in cases when conventional T2-weighted imaging fails, specially in thin and rapidly moving walls
Morphology and Function	bSSFP	Short-axis plane covering entire LV Long-axis - 3 slices each plane (2CH, 4CH and LVOT) Slice thickness = 6–8 mm Interslice gap = 2–4 mm	Mandatory for all CMR studies investigating TS. It will give information on the hallmark of the disease, regional abnormal contractility not related to coronary territory
Quantitative Tracking Techniques for Myocardial Motion and Strain	Myocardial Tissue Tagging (SPAMM or others) or a post-processing of regular bSSFP cine images	Short-axis plane covering entire LV Long-axis - 3 slices each plane (2CH, 4CH and LVOT) Slice thickness = 6–8 mm Interslice gap = 2–4 mm	Tagged or not tagged images require specific softwares for analysis. On bSSFP images is a novel technique with high potential for translating into routine clinical practice allowing tracking of tissue voxel motion of cine-CMR images to assess myocardial strain, velocities and displacement. Potentially useful for detection of subclinical cardiac involvement in TS, or previous TS in the recovery phase.
First-pass perfusion	Saturation-recovery imaging with bSSFP readout Gd contrast-first-pass bolus: 0.1 mmol/kg at 4–5 mL/s Immediately after – 2nd Gd bolus for LGE: + 0.1 mmol/kg	3–6 slices acquired in short axis plane of LV Slice thickness = 8 mm	Images at rest can help on identifying thrombus or previous chronic myocardial infarctions with replacement fibrosis.
EGE	<2 min after 2nd Gd bolus 2D segmented IR gradient echo-inversion time set at 500–550 ms at 1,5 T (identify thrombus). Single-shot or PSIR versions can be an alternative here. Less adopted, the traditional non-gated free-breathing T1w FSE images pre and post Gd bolus, with myocardial SI per se or in relation to skeletal muscle can be used.	Short-axis plane covering LV (especially mid-apical segments) Long-axis – 1 or more slice each plane (2CH, 4CH and LVOT) Slice thickness = 8 mm Interslice gap = 2	A surrogate for capillary leakage and hyperemia in the myocardium. Few data on the literature on the findings of these techniques in TS.
LGE	5–10 min after 2nd Gd bolus 2D segmented IR gradient echo with or without Phase-Sensitive IR (PSIR) Single-shot or 3D versions of LGE can also be used.	Short-axis plane covering LV (especially mid-apical segments) Long-axis – 1 or more slice each plane (2CH, 4CH and LVOT) Slice thickness = 8 mm Interslice gap = 2	The usual finding in TS is absence of significant myocardial LGE by visual analysis. Quantitative analysis using softwares with a variety of thresholds techniques can detect small amounts of patchy LGE.

*Gd* gadolinium

#### Contractile function

**Fig. 2:**
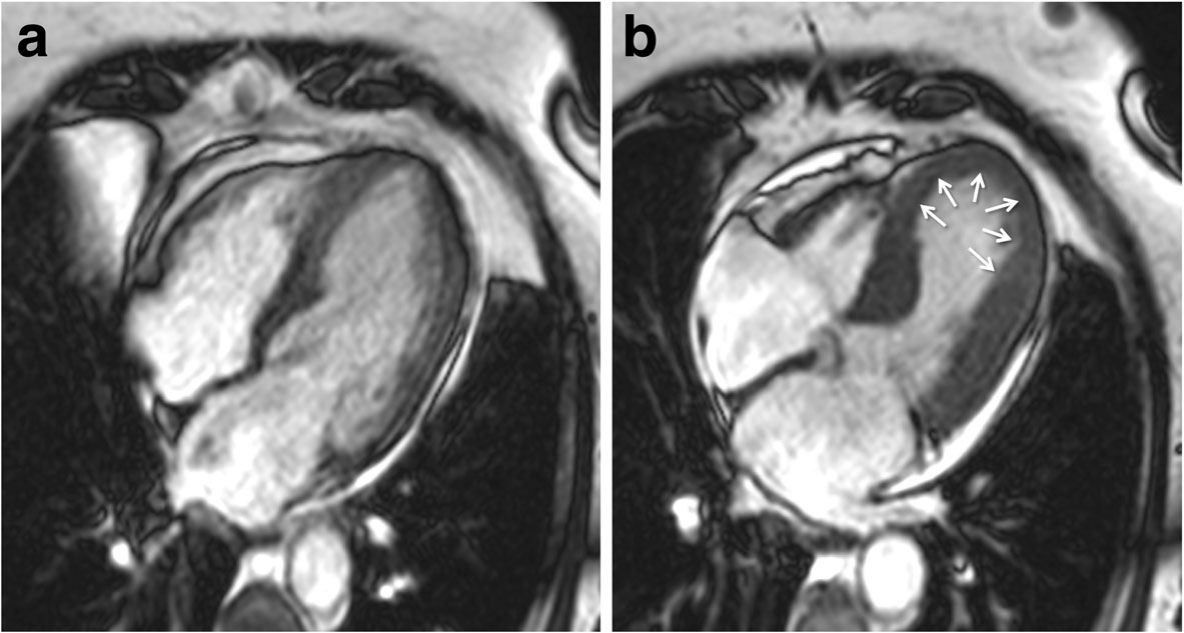
Typical apical balloning in takotsubo syndrome. Cine CMR 4-chamber view (**a**-late diastole; **b**-late systole). See Additional file 1

**Fig. 3:**
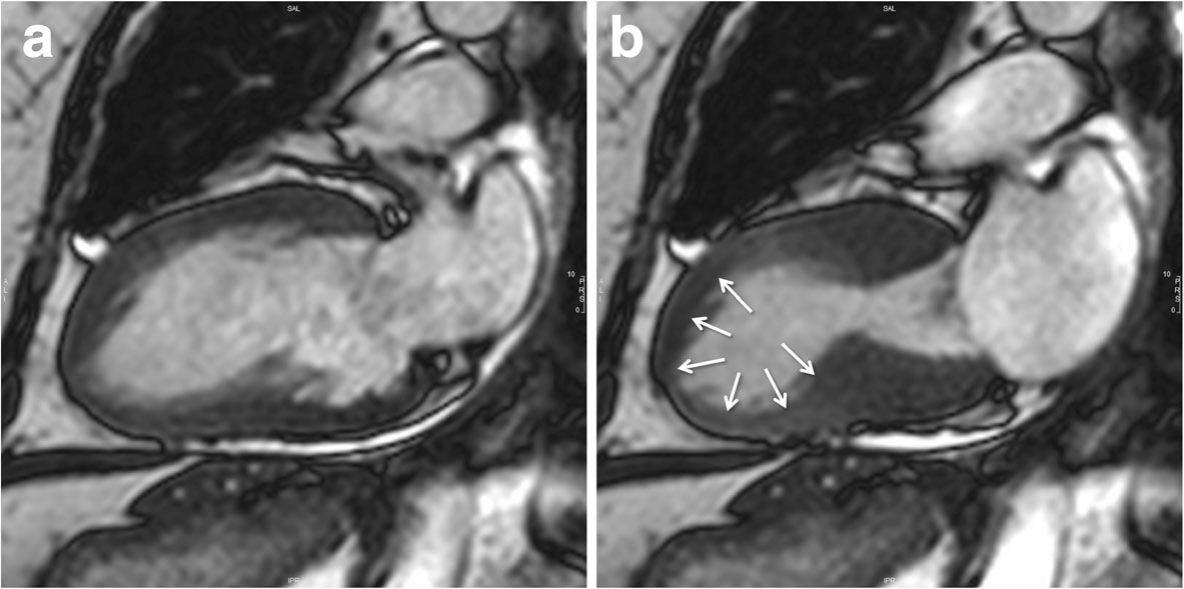
Typical apical balloning in takotsubo syndrome. Cine CMR 2-chamber view (**a**-late diastole; **b**-late systole). See Additional file 1

**Fig. 4:**
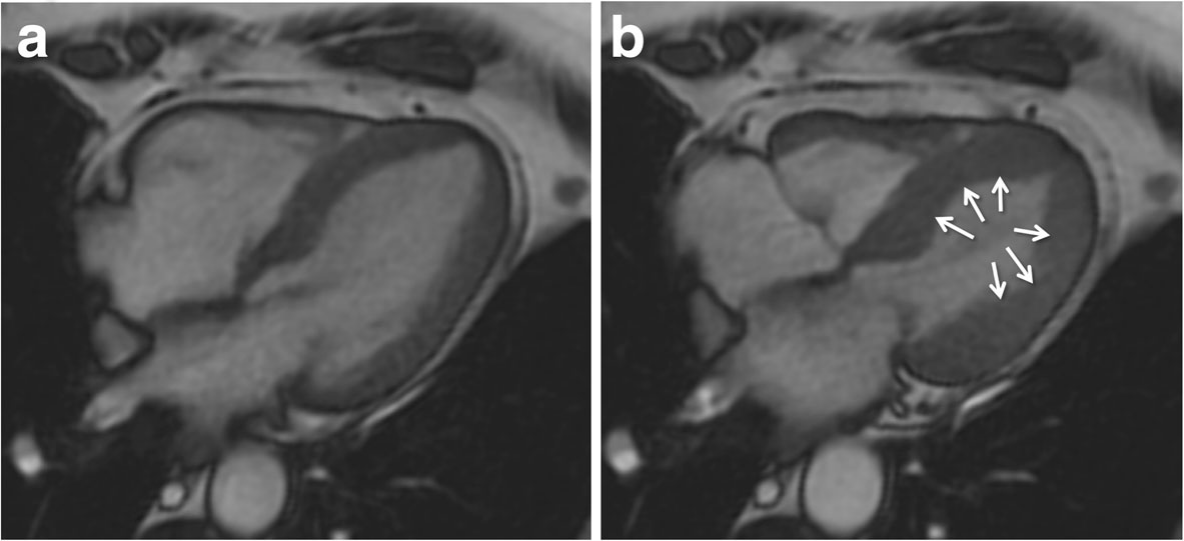
Mid-ventricular variant of takotsubo syndrome. Cine CMR 4-chamber view (**a**-late diastole; **b**-late systole). See Additional file 3

**Fig. 5:**
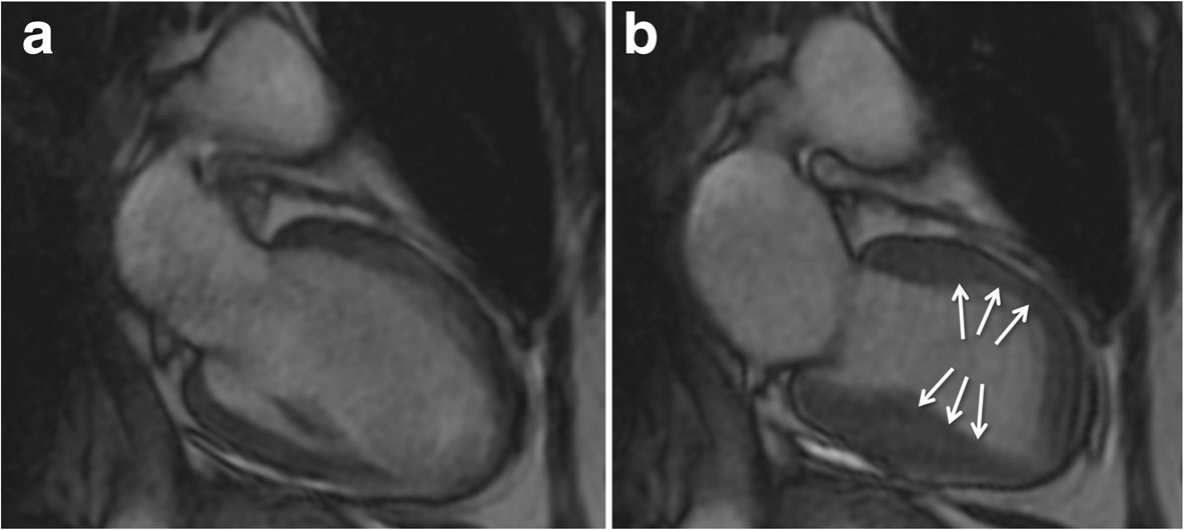
Mid-ventricular variant of takotsubo syndrome. Cine CMR 2-chamber view (**a**-late diastole; **b**-late systole). See Additional file 3



Cine sequences in CMR are a critical component in the diagnosis of TS and its variants. The word *takotsubo* means ‘octopus pot’ in Japanese, as the LV assumes a similar shape. The ‘typical form’ presents with contraction abnormalities in the ventricular wall, equally affecting the anterior, inferior, and lateral walls, that extend beyond a single epicardial vascular territory. This ‘circumferential pattern’ can be considered a hallmark of TS [12]. Another finding suggestive of TS is the presence of basal segments hyperkinesia, contributing to the characteristic morphology (Figs. 2 and 3; see Additional files 1 and 2). However, other contraction patterns during the acute phase of TS may be present. A recent report suggested that as many as 40 % of patients have a mid-ventricular variant [13], with mid-ventricular akinesis and apical sparing (Figs. 4 and 5; see Additional file 3). Basal akinesis with mid-ventricular and apical sparing also has been reported [14]. No differences in demographic, clinical, angiographic, laboratory parameters, or outcome were found between typical versus atypical TS. There are some cases reporting a dynamic pattern of wall motion abnormality in the same patient [15].

**Additional file 1:** Typical apical balloning in takotsubo syndrome. Cine CMR loops in 4- and 2-chamber view. (MOV 38365 kb)




**Additional file 2:** Typical apical balloning in takotsubo syndrome. Cine CMR loop in short-axis all slices multiview. Upper panels-basal slices. Middle panels-middle slices. Lower panels-apical slices. According to the colour-scale, red represents the most positive radial strain and dark-blue represents the most negative radial strain. (MOV 3530 kb)




**Additional file 3:** Mid-ventricular variant of takotsubo syndrome. Cine CMR loops in 4- and 2-chamber view. (MOV 21648 kb)


These aspects can be associated with a systolic ventricular dysfunction, severe in some patients, producing clinical symptoms and signs of heart failure.

**Fig. 6:**
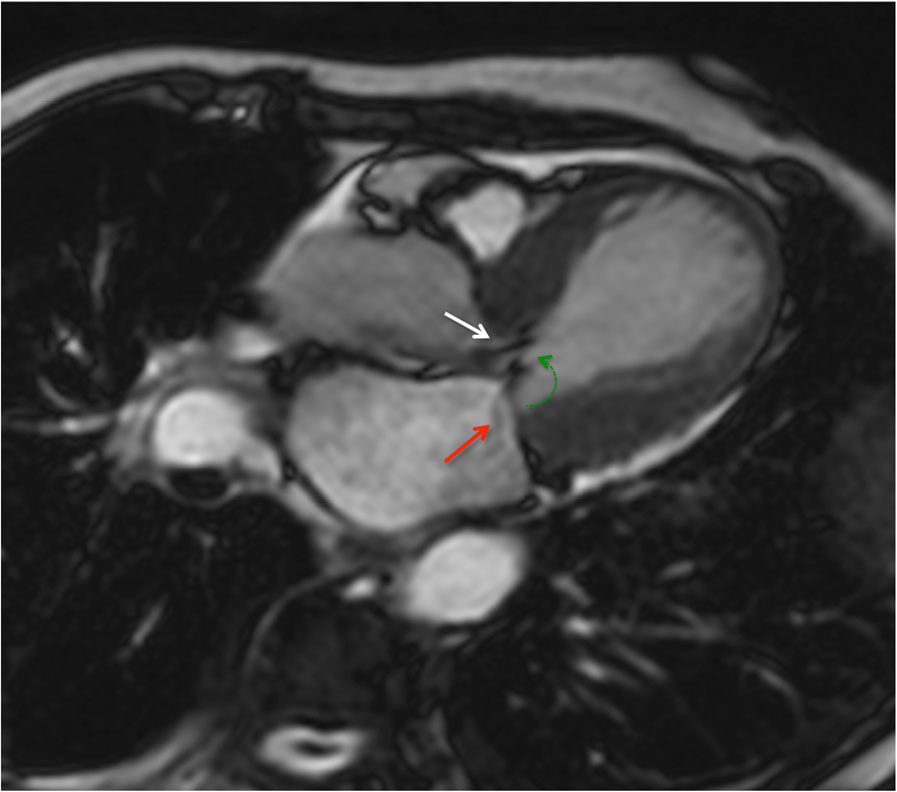
Cine CMR in horizontal long-axis view demonstrating the systolic “jet” in left ventricular outflow tract (*white arrow*) with associated systolic anterior motion of the mitral leaflets (*green arrow*) and functional mitral valve regurgitation (*red arrow*). This dynamic obstruction can develop as a result of dyskinetic apical and midventricular segments with hyperdynamic function of basal segments. See Additional file 4



Both basal hyperkinesis and mid-apical akinesis can produce a dynamic obstruction in the LV outflow tract associated or not with systolic anterior motion of the mitral leaflets and/or functional mitral valve regurgitation (Fig. 6; see Additional file 4). For an accurate measurement of mitral regurgitation volume and LV outflow tract gradient, phase contrast techniques can be used.

**Additional file 4:** Dynamic left ventricular outflow tract obstruction as a result of dyskinetic apical and midventricular segments with hyperdynamic function of basal segments. The cine CMR loop in horizontal long-axis view depicts the systolic “jet” in left ventricular outflow tract with associated systolic anterior motion of the mitral leaflets and functional mitral valve regurgitation. (MOV 33090 kb)


It is known that wall motion analysis with CMR has a pivotal role in clinical practice. At the present, image analysis is most commonly performed qualitatively. However, the diagnostic accuracy of qualitative assessment has been shown to be considerably operator dependant [16].

Feature/tissue tracking CMR is a novel technique that allows quantification of motion and strain using standard steady-state free-precession cine sequences of routine ventricular morpho-functional protocol. So far, quantitative data on regional myocardial function in TS have been explored by echocardiography. Heggemann et al. [17] described abnormal global and regional strain patterns during the acute phase of patients with TS that improved over time. Furthermore, subtle abnormalities of regional LV function seemed to persist into the early follow-up period as suggested by the presence of post-systolic shortening in more than half of LV segments. The authors concluded that long-term follow-up is needed to clarify whether these abnormalities will further improve.

**Fig. 7:**
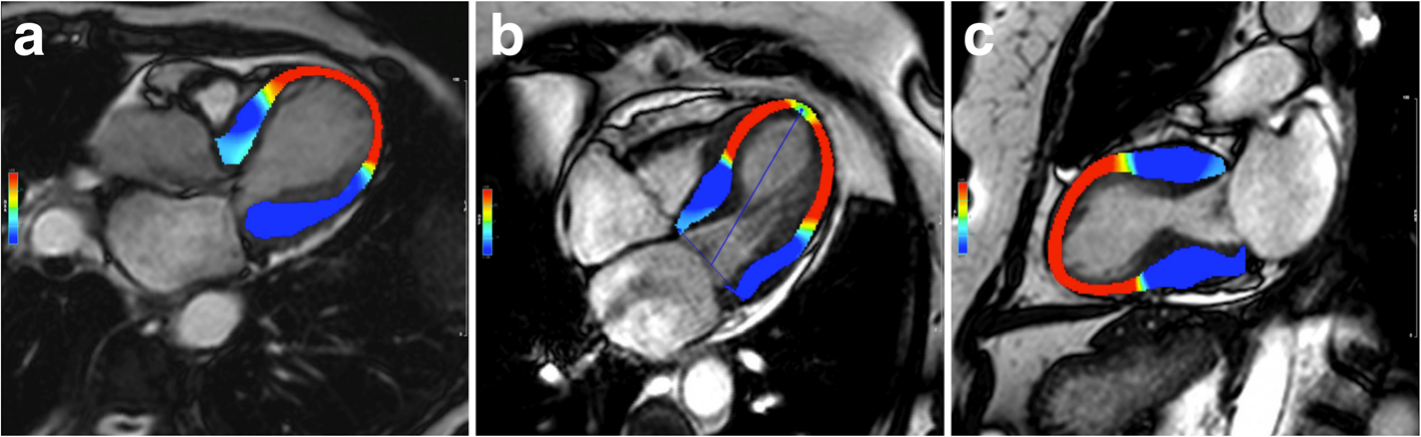
2-D longitudinal strain analysis by tissue-tracking CMR (Software CVI42 Version 5, Circle Cardiovascular Imaging Inc., Calgary, Canada) at end-systole in horizontal long-axis view (**a**), 4-chamber view and 2-chamber view (**c**) in a patient with the ‘classical form’ of takotsubo syndrome. According to the colour-scale (at the left on each panel), red represents the most positive longitudinal strain and dark-blue represents the most negative longitudinal strain (normal systolic longitudinal strain is negative representing shortening). At end-systole, mid-apical balloning is represented on red with a ‘circumferential pattern’. See Additional files 5, 6 and 7

**Fig. 8:**
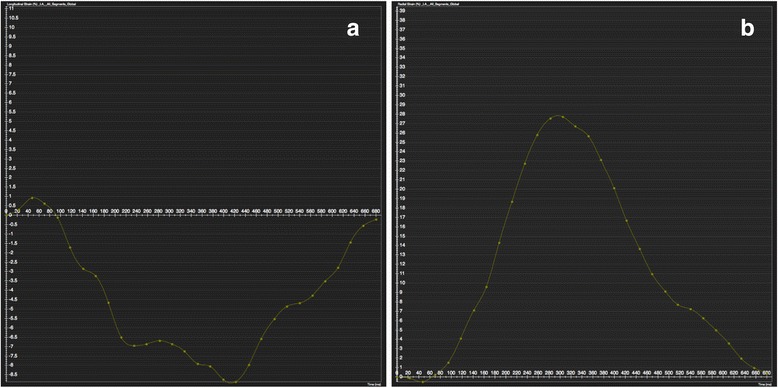
Global longitudinal (**a**) and radial (**b**) strain curves of the left ventricule by tissue-tracking CMR (Software CVI42 Version 5, Circle Cardiovascular Imaging Inc., Calgary, Canada)



Regarding feature/tissue tracking CMR, further studies are undoubtedly needed to determine its role in both clinical and research environments (Figs. 7 and 8; see Additional files 5, 6 and 7).

**Additional file 5:** 2-D longitudinal strain analysis by multimodality tissue-tracking CMR (Software CVI42 Version 5, Circle Cardiovascular Imaging Inc., Calgary, Canada) in 2-chamber view. (MOV 42270 kb)




**Additional file 6:** 2-D radial strain analysis by multimodality tissue-tracking CMR (Software CVI42 Version 5, Circle Cardiovascular Imaging Inc., Calgary, Canada) in short-axis all slices multiview). Upper panels-basal slices. Middle panels-middle slices. Lower panels-apical slices. According to the colour-scale, red represents the most positive radial strain and dark-blue represents the most negative radial strain. (MOV 55141 kb)




**Additional file 7:** Cine CMR loop of 4-D radial strain analysis (Software CVI42 Version 5, Circle Cardiovascular Imaging Inc., Calgary, Canada), depicting the typical apical balloning (blue) in a patient with takotsubo syndrome. (MOV 20707 kb)


#### Myocardial edema

An important hallmark of inflammatory cell injury is the increased permeability of cellular membranes. Whereas initial membrane defects are of functional nature, leading to Na + influx and subsequent intracellular edema, a more severe injury allows for a net efflux of water and transmembranous leakage of larger molecules such as troponin, eventually leading to loss of cellular functions.

Tissue T2 weighted (T2-W) imaging is a method that assesses magnetic image contrast, which is directly affected by the change in tissue water content, resulting in a high signal intensity of edematous tissue [18]. Black-blood T2-W triple-IR [19] provide contrast between regional edema and normal myocardium due to the dual suppression of the fat and flowing blood signal. When global myocardial involvement is suspected, a quantitative approach should be preferred, classically by calculating the SI ratio between myocardium and skeletal muscle − T2 SI ratio − and a cutoff value of ≥1.9 is considered significant, or by newer and promising parametric mapping techniques, especially T2 mapping.

**Fig. 9:**
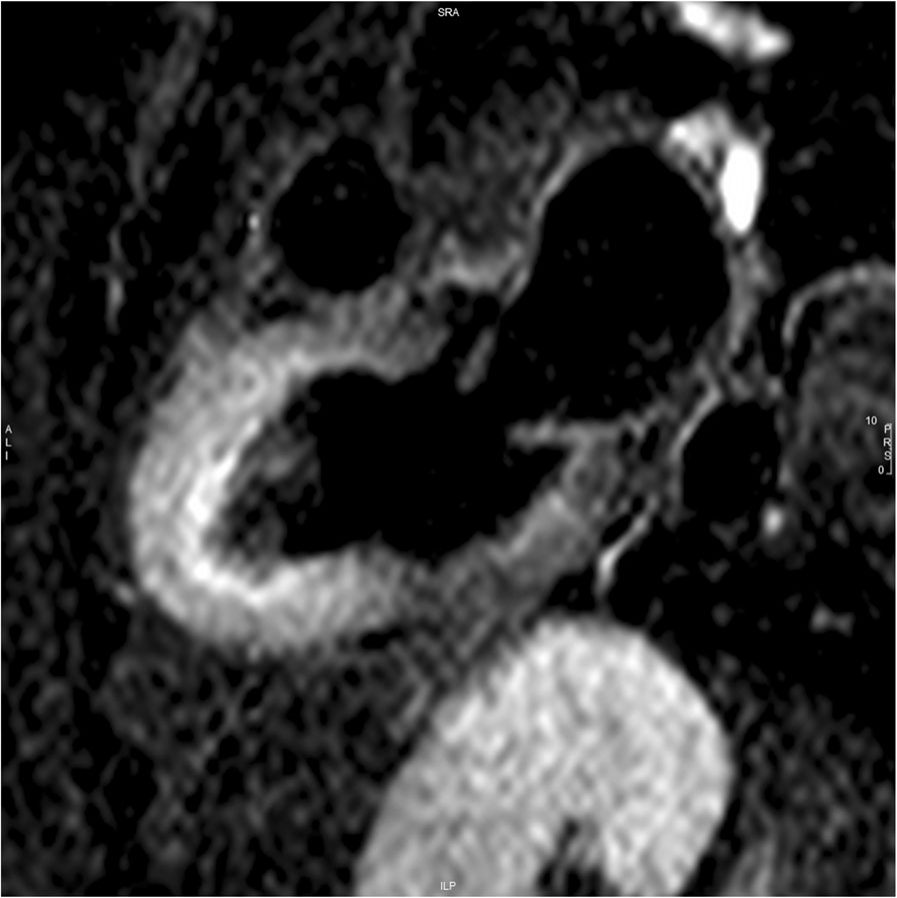
Myocardial edema. T2-weighted triple-inversion recovery 2-chamber view showing transmural signal hyperintensity in the mid-apical segments of left ventricle

**Fig. 10:**
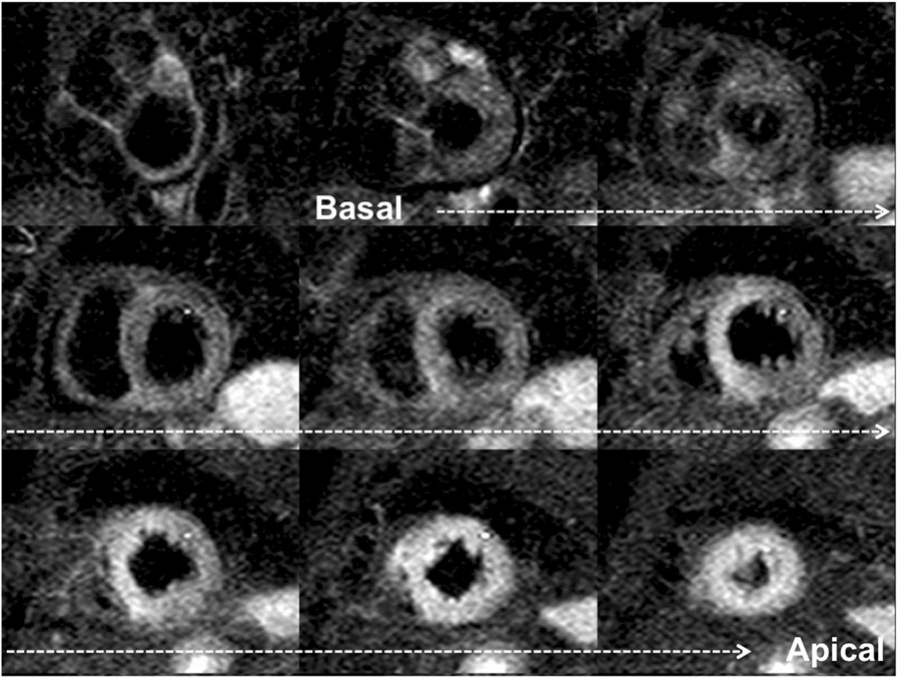
Myocardial edema. T2-weighted triple-inversion recovery short-axis view showing transmural signal hyperintensity in the mid-apical segments of left ventricle

In TS one of the most characteristic finding is myocardial edema, a direct result of the injury process. It assumes a diffuse or transmural distribution in both apical and mid planes, a location that is not restricted to a particular vascular territory. Those areas of edema typically match the dysfunctional ventricular contraction area observed with cine CMR sequences, which are usually globally circumferential, but restricted to a portion of the LV, such as involving all segments of apical portion, or alternatively mid or basal portions (Figs. 9 and 10).

These features can be used to differentiate TS from acute myocardial infarction, in which edema usually has a coronary distribution on the LV wall.

In acute myocarditis patients, T2-W triple IR sequence shows high signal intensity often with a multifocal and heterogeneous distribution in regions with LGE, that could be transmural, but frequently with higher signal intensity on mid-wall or subepicardial myocardial layer.

**Fig. 11:**
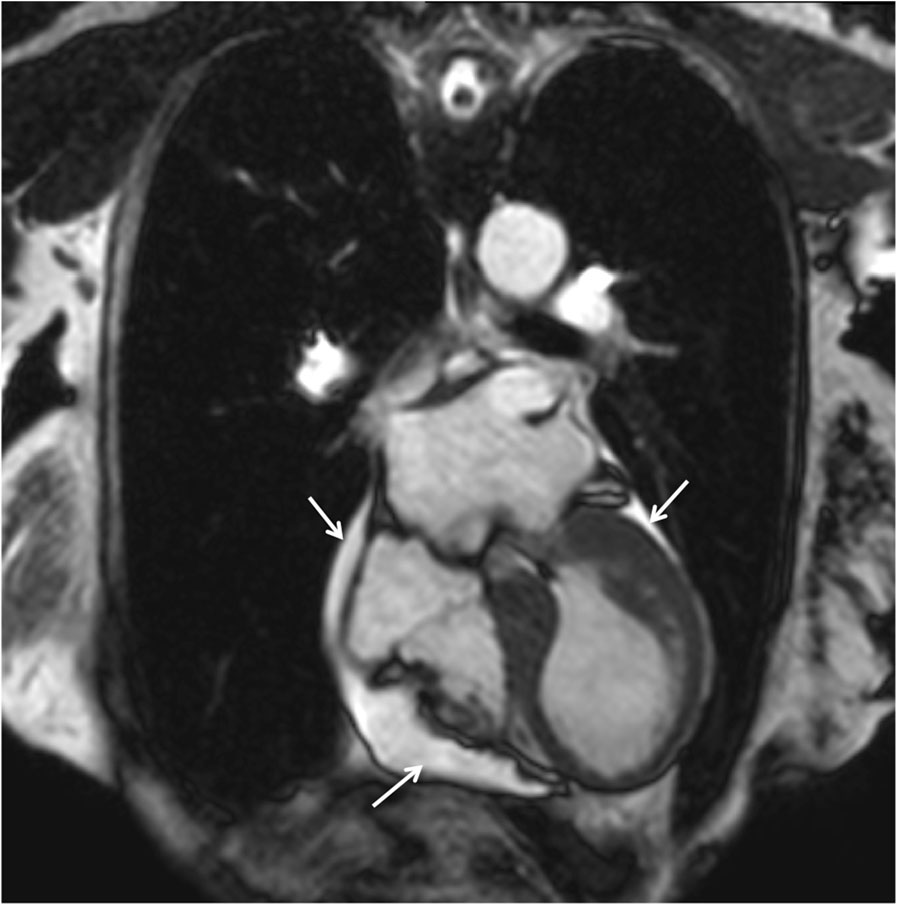
Pericardial effusion (arrows) in a patient with takotsubo syndrome. Cine CMR 4-chamber view (late systole)

**Fig. 12:**
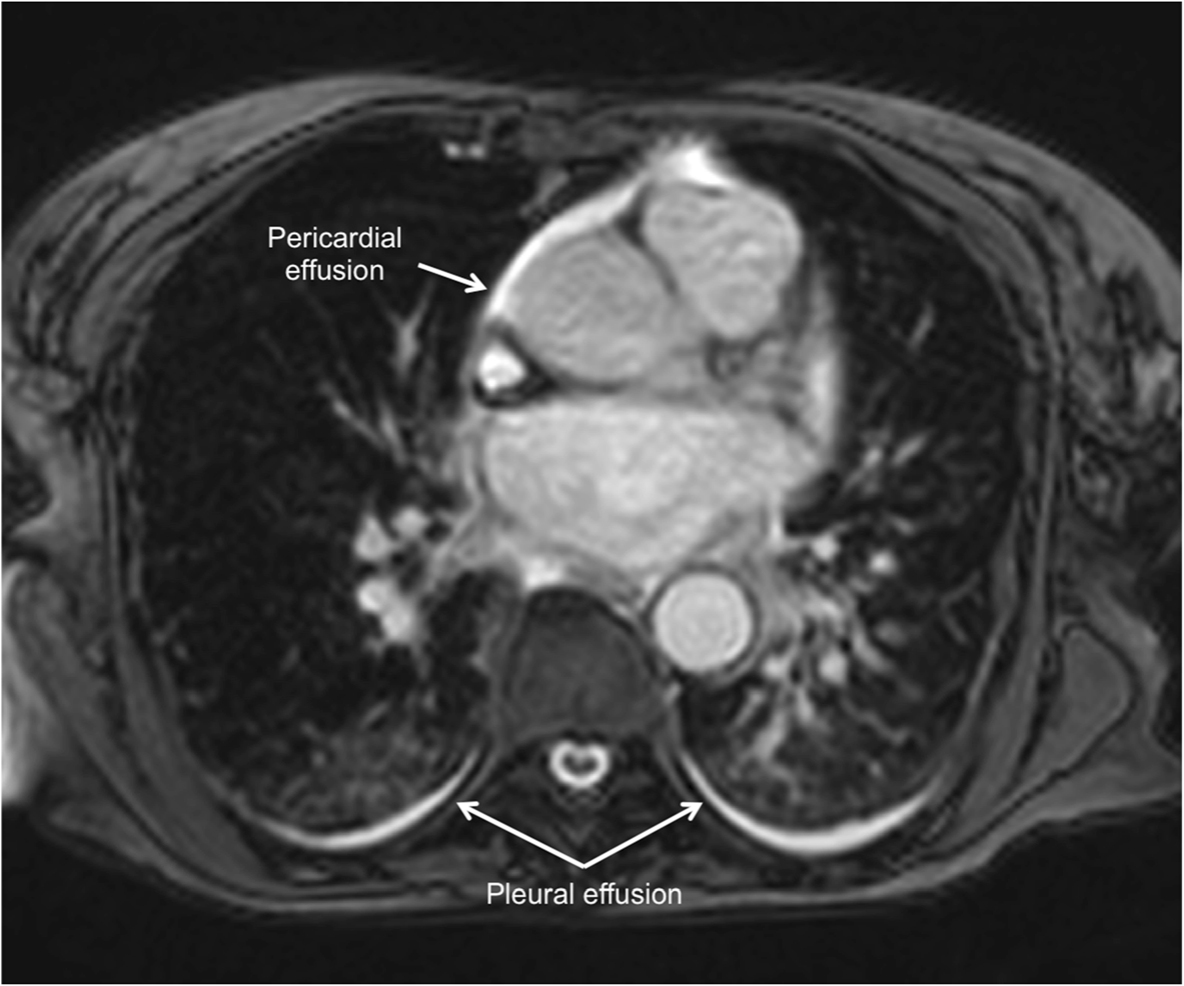
Pericardial and pleural effusions in a patient with takotsubo syndrome. Cine CMR axial view at the level of the pulmonary valve

Another indicator of the inflammatory processes in TS is the slight association between edema and pericardial effusion [20] (Figs. 11 and 12).

Initial T2 hyperintensity reduces over the following weeks, whereas T2 hyperintensity of segments affected by myocardial infarct is maintained for more than 2 or 3 months after symptom onset [21, 22].

To note, even though T2-W triple IR is normally used in CMR to detect edema, some authors have suggested the use of T2 mapping technique to diagnose it, which seems to be more insensitive to motion artifacts [23].

Despite increasing evidence on the use of T2-W triple IR, it is a recognized motion and blood pool artifact prone sequence. Parametric mapping techniques, especially T2 mapping, are emerging as a more robust alternative for edema appreciation and quantification in diverse clinical scenarios. Future research exploring the role of T2 mapping in TS is required.

#### Myocardial perfusion

In most cases, LV segments show no perfusion defects and findings on these techniques are usually normal in patients with TS. However, in some patients CMR first-pass perfusion studies may appear normal at basal segments but demonstrate subendocardial perfusion defects, especially more apically [24]. According to the most accepted mechanism of TS, which proposes a vigorous neurohumoral discharge precipitated by emotional stress, a severe microvascular dysfunction could be expected, leading to secondary myocardial stunning.

#### Thrombus assessment

EGE sequence, ideally performed within 2 min after contrast agent infusion, is optimal for identification of adherent endocardial thrombus on mid-apical segments with severe hypokinesis or akinesis. Thrombus is recognized as a low signal intensity (no gadolinium uptake), nearly black, that makes contrast with the intermediate signal of myocardium and blood pool, when de inversion time of the IR gradient echo sequence is set to 500–550 ms.

**Fig. 13:**
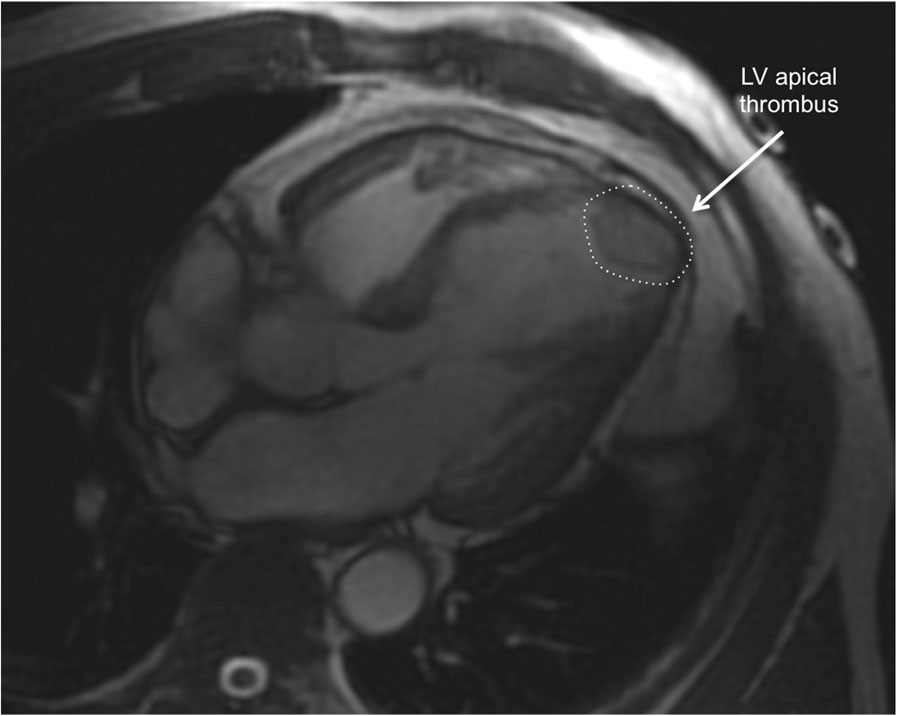
Apical thrombus (dashed circle), one of the complications of takotsubo syndrome. Cine CMR horizontal long-axis view

In some cases, LV thrombus can be detected by cine CMR (Fig. 13), although these sequences are less sensitive for this purpose.

#### Myocardial necrosis and fibrosis

LGE depends on differences in the volume of distribution of gadolinium in normal and pathologically altered myocardium [25] and several morphological changes within the tissue may contribute to a larger volume of distribution.

In general, LGE indicates the relative changes of the extracellular and intracellular volumes. Therefore, any changes to the interstitium, such as edema or fibrosis, increase the volume of distribution causing LGE [26]. Furthermore, myocardial necrosis causes intracellular accumulation of gadolinium, which contributes greatly to an expansion of the volume of distribution and LGE. Nonetheless, in edematous viable myocardium the ratio of the extracellular and intracellular volumes is not substantially altered [27]. Therefore, the peri-infarct zone involving the edematous viable myocardium but not the infarcted non-viable myocardium, does not exhibit significant LGE on CMR.

**Fig. 14:**
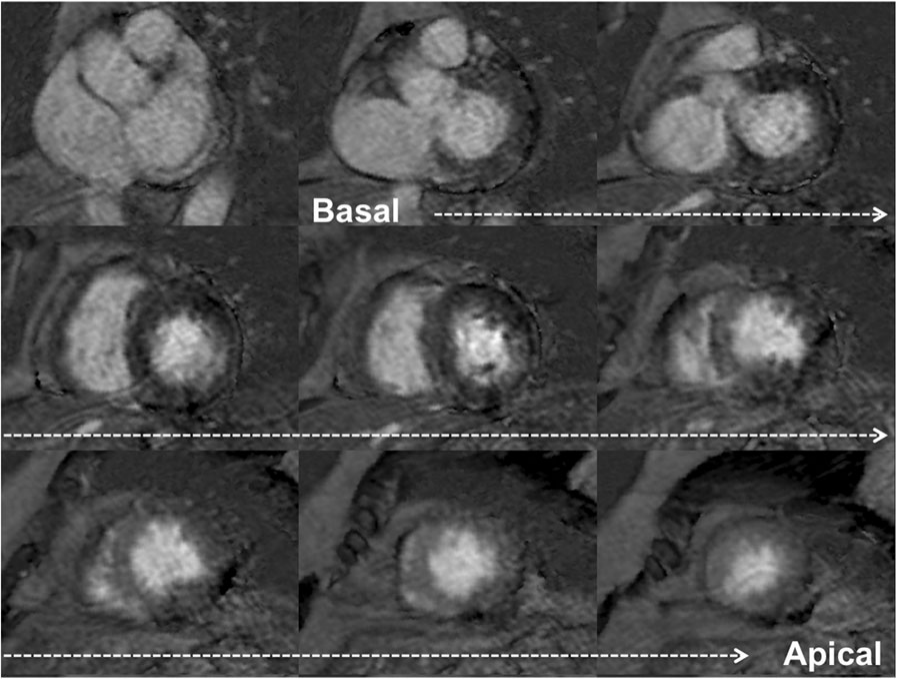
Late gadolinium enhancement CMR short-axis view depicting patchy higher signal intensity in the mid-apical segments of left ventricle, in a patient with takotsubo syndrome within 48 h of admission. These findings probably reflect a process of diffuse reactive fibrosis

The absence of LGE in TS patients as documented by CMR studies has been described in many cases and is a common diagnostic criterion in most CMR centers. However, several studies challenged this notion by reporting delayed hyperenhancement in TS patients [28, 29] (Fig. 14). Rolf et al. [30] described that the LGE signal intensity found in patients with TS within 24 h of admission was lower than that usually documented in cases of myocardial infarction or myocarditis and was no longer present on follow-up CMR 2 weeks later. Also the extent of LGE as represented by the relative mass of enhancing tissue is less than usually reported in studies of myocardial infarction [31]. More recently, in a large multicenter cohort study, Eitel et al. [32] showed that LGE was detected in only 9 % of patients when using a threshold of 3 standard deviation (SD) above the mean of remote myocardium to define significant enhancement. None of their patients had evidence of LGE when using a threshold of 5 SD, which is usually proposed as the cutoff for fibrosis detection in myocarditis and acute myocardial infarction. This points toward the fact that using different threshold for LGE detection in different studies may be responsible for the different prevalence of LGE-positive patients. The authors concluded that the absence of significant LGE (>5 SD) combined with myocardial edema and marked LV ballooning is a unique feature of TS.

Notwithstanding with this, a pathophysiological explanation for LGE in TS has been investigated. It is known that the level of extracellular matrix proteins is elevated and the amount of collagen-1 and fibronectin is significantly raised in the phase of severe contractile impairment, followed by normalization in functional recovery phase [33]. In the study by Rolf et al. [30] patients also underwent LV endomyocardial biopsy in the acute phase and after recovery, to determine the amount of extracellular matrix. All patients with TS showed a significant increase of collagen-1 compared with control tissue. Moreover, the amount of collagen-1 was significantly higher in LGE positive patients [defined as an area of signal intensity that was 2 SD or greater than the signal intensity of nonenhanced myocardium]. The latter group showed no increased levels of cardiac biomarkers, leading to the hypothesis that LGE cannot be attributed to differences in sarcolemmal integrity. That finding was supported by the fact that signs of oncotic cell death under electron microscopy were not present on tissue samples. Also, there were no differences in the extent of edema on T2 weighted measurements between groups. Therefore, evidence that LGE in TS is caused by either necrosis or edema is lacking, being plausible a process of transient fibrosis (or diffuse reactive fibrosis).

Differently, Sacha et al. [34] and Maréchaux et al. [35] reported cases of contraction-band necrosis detected at autopsy, a possible explanation for LGE in patients with TS.

Some authors investigated the clinical implications of LGE (using different thresholds) in TS. Eitel et al. [32] showed no relation between the occurrence of LGE and clinical presentation, mortality, age, sex, ECG pattern, or type of stress trigger in 158 patients with TS. Naruse et al. [36] reported, in a cohort of 20 patients with TS, no significantly differences in age, sex, congestive heart failure, peak creatine kinase, noradrenaline elevation, ECG abnormalities, ejection fraction, type of TS and time from onset of symptoms to CMR in the LGE positive group. However, there was a greater prevalence of cardiogenic shock (38 vs 0 %, *p* = 0,049) and longer time to normalize electro and echocardiographic changes. They also reported a positive correlation between the LGE area ratio (volume of enhanced tissue/total volume of LV myocardium) in the sub-acute phase (2–7 days after presentation) and the duration to normalization of both ECG (*r* = 0.738, *p* = 0.037) and echocardiogram (*r* =0.762, *p* = 0.028).

The utility of LGE is undeniable in the setting of focal myocardial fibrosis and necrosis, where different signal intensity threshold methods are used to detect enhancement. However, when myocardial injury is subtler and there are no confidently recognizable reference regions of normal myocardium, fibrosis may go undetected on conventional LGE threshold methods. In contrast, T1 mapping sequences provide a different approach to tissue characterization, permitting the measure of the intrinsic relaxation times of myocardium pre and post gadolinium infusion, not dependent on relative signal intensities. Therefore, it has the potential to improve the sensitivity of CMR tissue characterization, especially when diffuse and subtle fibrosis is the possible mechanism, like in TS.

### Right ventricular involvement in TS

An important issue that has recently been seen with the use of CMR and its cine sequences is a significant percentage of patients (up to one-third) with right ventricular involvement, which in turn was associated with longer hospitalization, worse markers of heart failure, particularly pleural effusion (Fig. 12) and older age. Consequently, biventricular ballooning may portend a more severe prognosis, when compared with isolated LV involvement [37, 38].

## Assessment of complications

Cardiovascular complications associated to TS are listed in Table 3. From this group of complications, most of them can be diagnosed and evaluated by CMR.

**Table 3:** Cardiovascular complications of TS

Complication	Study	Frequency	Comments/Prognostic implications
Pericardial effusion	Eitel I. et al. [20]	57,7 %	Rarely progresses to cardiac tamponade
Functional mitral valve regurgitation	Izumo M. et al. [39] Haghi D. et al. [40]	25,5 % 19 %	Conflicting data regarding long-term prognosis
Dynamic LVOT obstruction	De Baker O. et al. [41]	19 %	More frequent in older patients with the presence of septal bulging Gradient ≥ 40 mmHg is a high-risk factor associated to cardiogenic shock and functional mitral valve regurgitation
Heart failure (Killip-class 3/4 on admission)	Stiermaier T. et al. [42]	13,3 %	Independent predictor of long-term mortality
Right ventricular involvement	Kagyiama M. et al. [38] Becher T. et al. [43]	18,6 % 22,8 %	Conflicting data regarding long-term prognosis
Cardiogenic shock	Stiermaier T. et al. [42] Becher T. et al. [43]	10,8 % 15,9 %	Independent predictor of long-term mortality
Life-threatening arrhythmias	Stiermaier T. et al. [44]	13,5 %	Higher prevalence of subtle fibrosis/necrosis and lower LV ejection fraction on CMR Higher mortality at 1 year (44 vs 10 %)
Thrombus formation	de Gregorio C. et al. [45]	~2,5 %	No risk factors for thrombus formation were reported
Systemic embolism	de Gregorio C. et al. [45]	<1 %	Predictor of long-term mortality
LV free wall rupture	Kumar S. et al. [46]	<1 %	Risk factors: female gender, older age, persistent ST elevation, higher systolic BP and EF

## Follow-up

A complete clinical follow-up including CMR and/or echocardiography for confirmation of LV function recovery should be available 3–6 months after presentation (see Additional file 8). Since the underlying pathophysiology of TS is believed to be a form of myocardial stunning, recovery of normal systolic function is important to confidently make the diagnosis (Fig. 1). Despite the morphological changes, the absence of myocardial scar virtually guarantees normalization of function within weeks or a few months. Table 4 summarizes the recovery criteria assessed by CMR during the follow-up period.

**Table 4:** Recovery criteria during follow-up assessed by CMR in patients with TS

Normalization of LV ejection fraction.	
End-diastolic and end-systolic volume reduction.	
Mean T2 SI and EGE ratios reduction.	
No LGE (using a threshold of 5 SD).	



**Additional file 8:** Follow-up CMR of the Fig. 3 Patient (cine loops in 4- and 2-chamber view) demonstrating complete resolution of left ventricular dysfunction 3 months after the acute event. (MOV 20713 kb)


## Conclusion

Diagnosis of TS has important implications for clinical management at presentation and afterward. CMR provides incremental information and allows the detection of relevant functional and tissue changes useful for the diagnostic work up of TS. The combination of typical regional wall motion abnormalities and presence of reversible myocardial injury may be helpful in differentiation of TS from other etiologies in overlapping clinical presentations.

## Abbreviations

CMR: Cardiac magnetic resonance
EGE: Early gadolinium enhancement
LGE: Late gadolinium enhancement
LV: Left ventricular
MINOCA: Myocardial infarction with non-obstructive coronary arteries
T2-W: Tissue T2 weighted
TS: Takotsubo syndrome

## References

[1] Sato H, Tateishi H, Uchida T, Kodama K, Haze K, Hon M (1990). Takotsubo type cardiomyopathy due to multivessel spasm. Clinical aspect of myocardial injury: from ischemia to heart failure.

[2] Iga K, Hori K, Kitaguchi K (1991). Transient segmental asynergy of the left ventricle of patients with various clinical manifestations possibly unrelated to the coronary artery disease. Jpn Circ J.

[3] Bybee KA, Prasad A, Barsness GW (2004). Clinical characteristics and thrombolysis in myocardial infarction frame counts in women with transient left ventricular apical ballooning syndrome. Am J Cardiol.

[4] Villarroel AH, Vitola JV, Stier AL (2009). Takotsubo or stress cardiom- yopathy: role of nuclear cardiology using (123)I-MIBG. Expert Rev Cardiovasc Ther.

[5] Sharkey SW, Windenburg DC, Lesser JR (2010). Natural history and expansive clinical profile of stress (takotsubo) cardiomyopathy. J Am Coll Cardiol.

[6] Templin C, Ghadri JR, Diekmann J (2015). Clinical features and outcomes of takotsubo (stress) cardiomyopathy. N Engl J Med.

[7] Citro R, Rigo F, D’Andrea A (2014). Echocardiographic correlates of acute heart failure, cardiogenic shock, and in-hospital mortality in tako-tsubo cardiomyopathy. JACC Cardiovasc Imaging.

[8] Redfors B, Vedad R, Angerås O (2015). Mortality in takotsubo syndrome is similar to mortality in myocardial infarction—A report from the SWEDEHEART1 registry. Int J Cardiol.

[9] Schneider B, Athanasiadis A, Schwab J (2014). Complications in the clinical course of tako-tsubo cardiomyopathy. Int J Cardiol.

[10] Prasad A, Lerman A, Rihal CS (2008). Apical ballooning syndrome (Tako-Tsubo or stress cardiomyopathy): a mimic of acute myocardial infarction. Am Heart J.

[11] Eitel I, Behrendt F, Schindler K (2008). Differential diagnosis of suspected apical ballooning syndrome using contrast-enhanced magnetic resonance imaging. Eur Heart J.

[12] Citro R, Rigo F, Ciampi Q (2011). Echocardiographic assessment of regional left ventricular wall motion abnormalities in patients with Tako-Tsubo cardiomyopathy: comparison with anterior myocardial infarction. Eur J Echocardiogr.

[13] Kurowski V, Kaiser A, von Hof K (2007). Apical and midventricular transient left ventricular dysfunction syndrome (tako-tsubo cardiomyopathy): frequency, mechanisms, and prognosis. Chest.

[14] Reuss CS, Lester SJ, Hurst RT (2007). Isolated left ventricular basal ballooning phenotype of transient cardiomyopathy in young women. Am J Cardiol.

[15] Rodriguez F, Nathan AS, Navathe AS, Ghosh N, Shah PB (2014). Serial classic and inverted pattern Takotsubo cardiomyopathy in a middle-aged woman. Can J Cardiol.

[16] Paetsch I, Jahnke C, Ferrari VA (2006). Determination of interobserver variability for identifying inducible left ventricular wall motion abnormalities during dobutamine stress magnetic resonance imaging. Eur Heart J.

[17] Heggemann F, Hamm K, Kaelsch T (2011). Global and regional myocardial function quantification in Takotsubo cardiomyopathy in comparison to acute anterior myocardial infarction using two-dimensional (2D) strain echocardiography. Echocardiography.

[18] Aletras AH, Tilak GS, Natanzon A (2006). Retrospective determination of the area at risk for reperfused acute myocardial infarction with T2-weighted cardiac magnetic resonance imaging: histopathological and displacement encoding with stimulated echoes (DENSE) functional validations. Circulation.

[19] Simonetti OP, Kim RJ, Fieno DS (2001). An improved mr imaging technique for the visualization of myocardial infarction. Radiology.

[20] Eitel I, Lücke C, Grothoff M (2010). Inflammation in takotsubo cardiomyopathy: insights from cardiovascular magnetic resonance imaging. Eur Radiol.

[21] Assomull RG, Lyne JC, Keenan N (2007). The role of cardiovascular magnetic resonance in patients presenting with chest pain, raised troponin, and unobstructed coronary arteries. Eur Heart J.

[22] Fernandez-Perez GC, Aguilar-Arjona JA, de la Fuente GT (2010). Takotsubo cardiomyopathy: assessment with cardiac MRI. AJR Am J Roentgenol.

[23] Thavendiranathan P, Walls M, Giri S (2012). Improved detection of myocardial involvement in acute inflammatory cardiomyopathies using T2 mapping. Circ Cardiovasc Imaging.

[24] Elesber A, Lerman A, Bybee KA (2006). Myocardial perfusion in apical ballooning syndrome correlate of myocardial injury. Am Heart J.

[25] Arheden H, Saeed M, Higgins CB (1999). Measurement of the distribution volume of gadopentetate dimeglumine at echo-planar MR imaging to quantify myocardial infarction: comparison with 99mTc-DTPA autoradiography in rats. Radiology.

[26] Kim DH, Choi SI, Chang HJ (2006). Delayed hyperenhancement by contrast-enhanced magnetic resonance imaging: clinical application for various cardiac diseases. J Comput Assist Tomogr.

[27] Li G, Xiang B, Dai G (2005). Tissue edema does not change gadolinium-diethylenetriamine pentaacetic acid (Gd-DTPA)-enhanced T1 relaxa- tion times of viable myocardium. J Magn Reson Imaging.

[28] Abdel-Aty H, Cocker M, Friedrich MG (2009). Myocardial edema is a feature of Tako-Tsubo cardiomyopathy and is related to the severity of systolic dysfunction: insights from T2-weighted cardiovascular magnetic resonance. Int J Cardiol.

[29] Haghi D, Fluechter S, Suselbeck T (2005). Delayed hyperenhancement in a case of Takotsubo cardiomyopathy. J Cardiovasc Magn Reson.

[30] Rolf A, Nef HM, Möllmann H (2009). Immunohistological basis of the late gadolinium enhancement phenomenon in tako-tsubo cardiomyopathy. Eur Heart J.

[31] Bogaert J, Kalantzi M, Rademakers FE (2007). Determinants and impact of microvascular obstruction in successfully reperfused ST-segment elevation myocardial infarction. Assessment by magnetic resonance imaging. Eur Radiol.

[32] Eitel I, von Knobelsdorff-Brenkenhoff F, Bernhardt P (2011). Clinical characteristics and cardiovascular magnetic resonance findings in stress (takotsubo) cardiomyopathy. JAMA.

[33] Nef HM, Möllmann H, Kostin S (2007). Tako-Tsubo cardiomyopathy: intraindividual structural analysis in the acute phase and after functional recovery. Eur Heart J.

[34] Sacha J, Maselko J, Wester A (2007). Left ventricular apical rupture caused by takotsubo cardiomyopathy–comprehensive pathological heart investigation. Circ J.

[35] Maréchaux S, Fornes P, Petit S (2008). Pathology of inverted Takotsubo cardiomyopathy. Cardiovasc Pathol.

[36] Naruse Y, Sato A, Kasahara K (2011). The clinical impact of late gadolinium enhancement in Takotsubo cardiomyopathy: serial analysis of cardiovascular magnetic resonance images. J Cardiovasc Magn Reson.

[37] Haghi D, Athanasiadis A, Papavassiliu T (2006). Right ventricular involvement in takotsubo cardiomyopathy. Eur Heart J.

[38] Kagiyama N, Okura H, Tamada T (2016). Impact of right ventricular involvement on the prognosis of takotsubo cardiomyopathy. Eur Heart J Cardiovasc Imaging.

[39] Izumo M, Nalawadi S, Shiota M (2011). Mechanisms of acute mitral regurgitation in patients with takotsubo cardiomyopathy: an echocardiographic study. Circ Cardiovasc Imaging.

[40] Haghi D, Röhm S, Suselbeck T (2010). Incidence and clinical significance of mitral regurgitation in Takotsubo cardiomyopathy. Clin Res Cardiol.

[41] De Backer O, Debonnaire P, Gevaert S (2014). Prevalence, associated factors and management implications of left ventricular outflow tract obstruction in takotsubo cardiomyopathy: a two-year, two-center experience. BMC Cardiovasc Disord.

[42] Stiermaier T, Moeller C, Oehler K (2016). Long-term excess mortality in takotsubo cardiomyopathy: predictors, causes and clinical consequences. Eur J Heart Fail.

[43] Becher T, El-Battrawy I, Baumann S (2016). Characteristics and long-term outcome of right ventricular involvement in Takotsubo cardiomyopathy. Int J Cardiology.

[44] Stiermaier T, Eitel C, Denef S (2015). Prevalence and Clinical Significance of Life-Threatening Arrhythmias in Takotsubo Cardiomyopathy. J Am Coll Cardiol.

[45] de Gregorio C, Grimaldi P, Lentini C (2008). Left ventricular thrombus formation and cardioembolic complications in patients with Takotsubo-like syndrome: a systematic review. Int J Cardiol.

[46] Kumar S, Kaushik S, Nautiyal A (2011). Cardiac rupture in takotsubo cardiomyopathy: a systematic review. Clin Cardiol.

